# Interaction between serum FGF-23 and PTH in renal phosphate excretion, a case-control study in hypoparathyroid patients

**DOI:** 10.1186/s12882-020-01826-5

**Published:** 2020-05-12

**Authors:** Forough Saki, Seyed Reza Kassaee, Azita Salehifar, Gholam Hossein Ranjbar Omrani

**Affiliations:** Shiraz Endocrinology and Metabolism Research Center, Shiraz University of Medical Sciences, P.O. Box: 71345-1744, Shiraz, Iran

**Keywords:** FGF-23, FE PO_4_, Hypoparathyroidism, PO_4_

## Abstract

**Background:**

phosphate homeostasis is mediated through complex counter regulatory feed-back balance between parathyroid hormone, FGF-23 and 1,25(OH)2D. Both parathyroid hormone and FGF-23 regulate proximal tubular phosphate excretion through signaling on sodium- phosphate cotransporters II_a_ and II_c_. However, the interaction between these hormones on phosphate excretion is not clearly understood. We performed the present study to evaluate whether the existence of sufficient parathyroid hormone is necessary for full phosphaturic function of FGF-23 or not.

**Methods:**

In this case-control study, 19 patients with hypoparathyroidism and their age- and gender-matched normal population were enrolled. Serum calcium, phosphate, alkaline phosphatase,parathyroid hormone, FGF-23, 25(OH)D, 1,25(OH)2D and Fractional excretion of phosphorous were assessed and compared between the two groups, using SPSS software.

**Results:**

The mean serum calcium and parathyroid hormone level was significantly lower in hypoparathyroid patients in comparison with the control group (*P* < 0.001 and *P* < 0.001, respectively). We found high serum level of phosphate and FGF-23 in hypoparathyroid patients compared to the control group (*P* < 0.001 and *P* < 0.001, respectively). However, there was no significant difference in Fractional excretion of phosphorous or 1,25OH2D level between the two groups. There was a positive correlation between serum FGF-23 and Fractional excretion of phosphorous just in the normal individuals (*P* < 0.001, *r* = 0.79).

**Conclusions:**

Although the FGF-23 is a main regulator of urinary phosphate excretion but the existence of sufficient parathyroid hormone is necessary for the full phosphaturic effect of FGF-23.

## Background

Phosphorus (PO_4_) has several biologic role in human, and is an essential ion in bone mineral component, cell membrane structure, and energy exchange. Also, it is a second messenger in controlling cellular biochemical activities through phosphorylation or dephosphorylation [1–3]. Kidney plays an important role in PO_4_ homeostasis. About 80% of the filtered PO_4_ is reabsorbed through specific sodium- phosphate cotransporters (NaPi II_a_ and II_c_) located in the proximal tubule [4–8].

PO_4_ serum concentration is kept within the normal range by a complex regulation between intestinal absorption, renal filtration- reabsorption, and bone resorption of PO_4_ mediated by regulatory hormones [9–11]. The most important hormones that regulate tubular PO_4_ handling, are parathyroid hormone (PTH) secreted by the parathyroid gland, and fibroblast growth factor 23 (FGF-23), which is an osteocytes derived hormone. FGF-23 decreases serum PO_4_ by inhibiting renal PO_4_ reabsorption through FGF-23-Klotho (coreceptor) signaling on NaPi II_a_ and II_c_ at proximal tubule of the kidney. FGF-23 also suppresses 1,25-dihydroxyvitamin D (1,25(OH)2 D) production by decreasing 1α-hydroxylase expression [12–14]. PTH promotes PO_4_ excretion by suppressing NaPi-II in the kidney [15]. PTH also enhances calcium absorption through the direct effect on bones and kidneys, and indirectly increases intestinal calcium and PO_4_ absorption via the stimulation of 1α -hydroxylase activity and increase 1,25(OH)2 D production [16–18]. The same site of action of FGF-23 and PTH in NaPi II_a_ and II_c_ at proximal tubule of kidney might raise the question that whether there is an overlapping effect between these hormones or not?

Hypoparathyroidism is a rare endocrine disorder characterized by inappropriately low or absent levels of PTH associated with hypocalcemia and hyperphosphatemia [17, 19]. Hypoparathyroidism might occur as a primary congenital defect or might be due to a secondary cause. The most common cause of secondary hypoparathyroidism is the incidental destruction of parathyroid glands during anterior neck surgeries. Other causes are autoimmune disorders, radiation to the neck and infiltrative disorders of the parathyroid glands [20–22].

In the present study, we aim to evaluate the role of FGF-23 on PO_4_ hemostasis in state of low or insufficient PTH in human. Hence, we conducted this case – control study to evaluate whether the renal excretion of PO_4_ by serum FGF-23 in patients with hypoparathyroidism was different from normal population or not.

## Methods

### Patients and method

A total of 38 participants including 19 patients with hypoparathyroidism and their healthy controls were enrolled in this study. The study was performed at Shiraz University of Medical Sciences affiliated endocrine clinics in Fars province, southern Iran, from October 2017 till March 2018. Both groups were matched for age and gender. Hypoparathyroidism was diagnosed on the basis of hypocalcemia (serum calcium less than 8.5 mg/dl) accompanied with documented PTH levels below the lower limit of the normal range.

All hypoparathyroid patients were follow up by an expert endocrinologist. Patients received proper doses of calcium carbonate (500 mg tablet, manufactured Toliddaru pharmaceutical, Tehran, Iran), and calcitriol (0.25 μg capsule, manufactured Zahravi pharmaceutical, Tehran, Iran) to maintain albumin-corrected serum calcium in the low-normal range (8–9 mg/dl) [23]. The exclusion criteria in both groups were renal failure (Glomerular filtration rate less than 60 ml/min), liver failure, other metabolic bone disease (e.g., rickets), hyperthyroidism, and diabetes mellitus. None of the patients received phosphate binder resins during the study.

### Laboratory tests

All the samples were taken after 8 h overnight fasting. Blood samples were centrifuged for 15 min at *3000 rpm* and the plasma was collected and stored at − 70 °C till further analysis. All the biochemical studies were performed at the endocrinology and metabolism research center laboratory of Shiraz University of Medical Sciences. Colorimetric assays were used to measure calcium (mg/dL), phosphorus (mg/dL), albumin (g/dL) and alkaline phosphatase (ALP) (IU/L) levels, by using Biosystem SA auto-analyzer, made in Spain. Serum PTH (pg/ml) and 25(OH)D (ng/ml) levels were assessed by Electrochemiluminescence methods produced by Roche company in Germany with Sensitivity, intra- and inter-assay CVs 3.3 and 5.1%, respectively. ELISA method was used to determine the serum intact FGF-23 (pg/ ml) and 1,25(OH)2D (pmol/l) using Bioassay technology laboratory kit. Intra- and inter-assay CVs for 1,25(OH)2D and FGF-23 were < 8 and < 10%, respectively. Normal references for serum calcium, phosphorus, ALP, PTH, 25(OH)D and 1,25(OH)2D were 8.5–10.5 mg/ dL, 3.5–5.5 mg/ dL, 44–147 IU*/*L, 10–65(pg/ml), 20–100 ng/ml, and 20 to 45 pg/ml, respectively. Initial morning urine collection was done to determine renal PO_4_ clearance. Urinary PO_4_ and creatinine concentrations were determined by digital flame spectrophotometer. Fractional excretion of phosphorous (FE PO_4_) was done using the following formula: FE PO_4_ = [PO_4_ (Urine) **×** Creatinine (Serum)] / [PO_4_ (Serum) **×** Creatinine (Urine)] **×** 100.

### Ethical statement

An informed written consent form was obtained from the participants after explaining the aim, method and goal of the study. Shiraz University of Medical Sciences local Ethics Committee and Vice-Chancellor of research at SUMS approved this study with number 1396-01-01-15,805.

### Statistics

SPSS statistical software (version 22, IBM) were used to perform Statistical analysis. Data are mentioned as mean ± SD. Shapiro-Wilk was used to evaluate the normality of data distribution. Normally distributed data were compared using Student’s *t-*test, and the Mann–Whitney test was used to compare non-normally distributed ones. Pearson’s test and Spearman’s ranking test were used to evaluate the correlations between normally distributed parameters and non-normal distributed ones, respectively. *P* value less than 0.05 was considered to be statically significant.

## Results

A total of 38 participants were enrolled in this study, 19 with hypoparathyroidism as case group and 19 volunteers with normal parathyroid function as the control group. Mean age in the case and control groups was 43.6 ± 17 years and 46.7 ± 15.9 years, which was not statistically significant (*P* = 0.57). Both case and control groups included 5 male and 14 female. Also, there were no significant differences in weight and BMI between case and control groups.

In the case group, 9 patients had hypoparathyroidism due to previous neck thyroidectomy and 10 patients were case of primary hypoparathyroidism. General characteristic of patients and controls are summarized in Table 1.

**Table 1:** General characteristics and biochemical studies in both case and control groups and the related comparisons

Variable	control	case	*P* value
**Age (y)**	46.72 ± 15.89	43.68 ± 17.01	0.57
**Weight (Kg)**	70.89 ± 14.33	75.06 ± 22.11	0.51
**Height (cm)**	159.83 ± 10.4	163.47 ± 9.92	0.29
**BMI (Kg/m**^**2**^**)**	27.82 ± 3.53	27.75 ± 7.83	0.97
**PTH (pg/ml)**	57.97 ± 18.06	8.92 ± 4.38	< 0.001
**Ca (mg/dl)**	9.20 ± 0.46	7.98 ± 0.86	< 0.001
**PO**_**4**_**(mg/dl)**	3.82 ± 0.46	5.26 ± 0.93	< 0.001
**ALP(IU/L)**	148.22 ± 37.9	132.58 ± 40.69	0.23
**1,25(OH)2D (pg/ml)**	23.73 ± 17.90	30.57 ± 17.77	0.25
**25 (OH)D (ng/ml)**	33.56 ± 32.35	53.11 ± 40.71	0.11
**FGF23 (pg/ml)**	24.66 ± 17.73	47.88 ± 22.14	< 0.001
**FE PO**_**4**_**(%)**	15.77 ± 6.64	16.96 ± 11.40	0.70

*BMI* Body mass index, *FGF-23* Fibroblast Growth Factor 23, *ALP* Alkaline phosphatase, *PO*_*4*_ phosphorus, *Ca* Calcium, *PTH* Parathyroid Hormone, *FE PO*_*4*_ Fraction excretion of phosphorus

The mean serum calcium and PTH level was significantly lower in the case group in comparison with the control group (*P* < 0.001 and *P* < 0.001, respectively). In patients with hypoparathyroidism serum PO_4_ was significantly higher than the control group (*P* < 0.001). Serum FGF-23 was higher in patients with hypoparathyroidism in comparison with the control group (*P* = 0.001). However, there was no significant difference between the case and control groups with respect to the mean serum level of 1,25(OH)2D, 25(OH)D, ALP, and FE PO_4_ (*P* = 0.25, *P* = 0.11, *P* = 0.23 and *P* = 0.08, respectively).

**Fig. 1:**
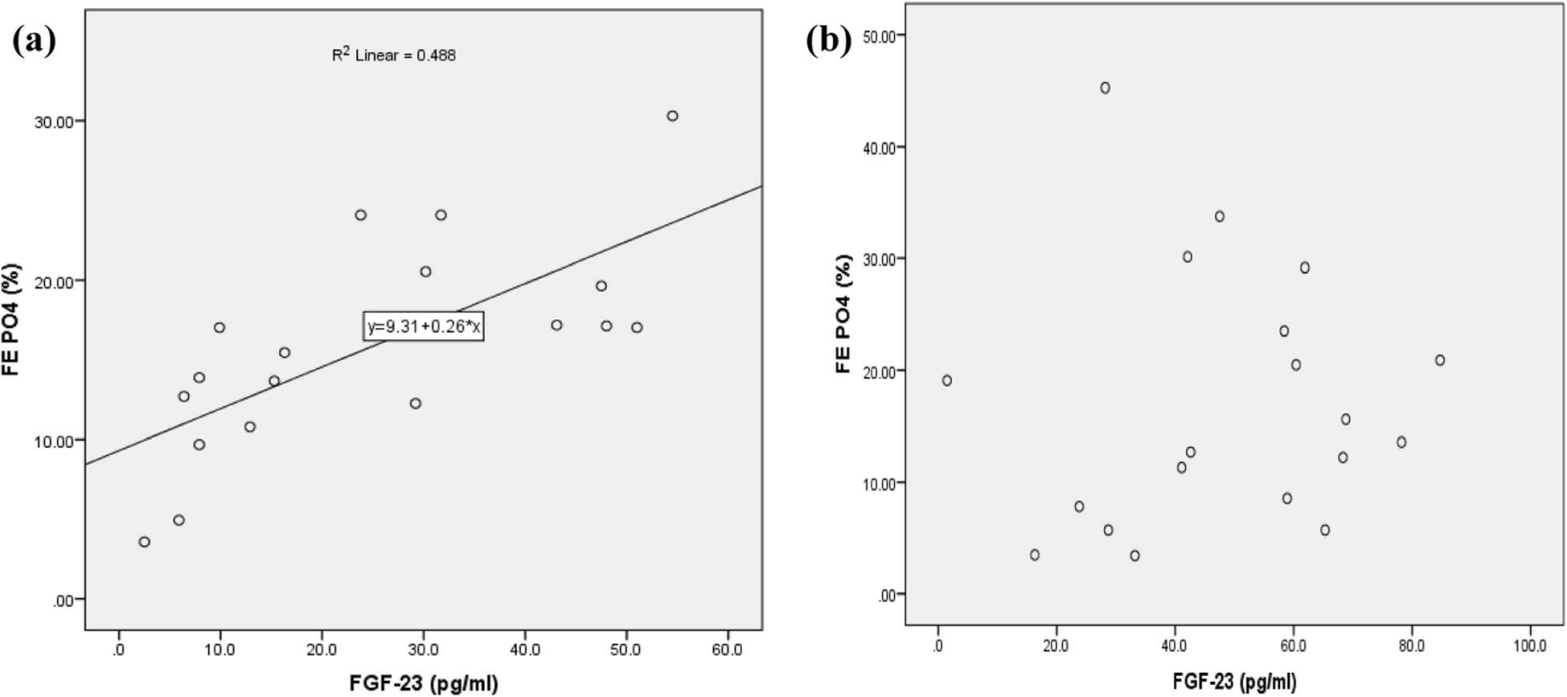
The correlation between values of serum FE PO_4_ and FGF-23 in control group (**a**) and hypoparathyroid cases (**b**)

As shown in Table 2 there was a strong positive correlation between FGF-23 and FE PO_4_ in the control; however, this correlation was not observed amongst hypoparathyroid patients. There was no correlation between FE PO_4_, serum PO_4_, PTH and 1, 25(OH)2 D in both case and control groups. Figure 1 shows the correlation between values of FE PO_4_ and serum FGF-23 in both control and case groups (Spearman rho =0.79, *P* < 0.001). In addition, we have done an analysis in our hypoparathyroid patients evaluating the association of FGF23 and serum calcium in 2 separate group of low calcium and normal calcium level. It showed that there was no correlation between calcium and FGF23 in low calcium and normal calcium level groups (*P* = 0.598 and *P* = 0.054, respectively).

**Table 2:** Correlation between fractional excretion of PO_4_ and serum biochemical parameters in the case and control groups, separately

		FGF-23	PO_4_	PTH
**FE PO**_**4**_	**Control group**	*P* < 0.001	*P* = 0.94	*P* = 0.97
		cc = 0.79	cc = 0.016	cc = 0.01
	**Case group**	*P* = 0.38	*P* = 0.82	*P* = 0.98
		cc = − 0.21	cc = − 0.053	cc = 0.005

## Discussion

Maintaining serum PO_4_ homeostasis necessitates a complex counter regulatory feed-back balance between PTH, FGF-23 and 1,25(OH)2 D [24–26]. FGF-23 and PTH are probably the most important phosphaturic hormones in human [1]. FGF-23 is mainly produced by osteoblasts and osteocytes. Local expression of FGF-23 coreceptor (Klotho) is necessary for its function at the renal proximal tubules [27]. It inhibits renal Phos reabsorption through inhibitory effects on NaPi II_a_ and II _c_ at proximal renal tubules [28]. However, there are still controversies about the action site of FGF-23 in the kidney [29]. previous studies had showed that klotho is essentially expressed in distal renal tubules, and alteration in the extracellular signal-regulated kinase (ERK) phosphorylation in distal tubules occurs soon after FGF-23 injection [30, 31]. Therefore, it still remain unclear as how FGF-23 could affect proximal tubules to suppress phosphate reabsorption. Data suggest that FGF-23 might require other factors such as PTH for signal transduction pathway at the proximal tubules [32].

PTH also increases renal PO_4_ excretion at proximal tubule of the kidney by reducing apical membrane NaPi II_a_ and II_c_ [14, 33, 34]. Moreover, PTH increases FGF-23 gene expression [35]. In addition to kidneys, parathyroid gland also express considerable amount of klotho and FGF-23 receptor [36]. On the other hand, FGF-Klotho complex could activate the MAPK pathway leading to decreased PTH mRNA and PTH secretion [37–39]. Olena et al. showed that the phosphaturic actions of PTH, are blunted by FGF-23 or Klotho deficiency. Hence, FGF-23 might be an important modulator of PTH signaling in the kidney [40].

Although the regulatory counterbalance between FGF-23 and PTH secretion was investigated, there is still insufficient information about the role of PTH on phosphaturic function of FGF-23 in human. Study of phosphaturic effect of FGF-23 in normal human physiology might be confounded by the fact that PTH and FGF-23 have some overlapping effects on PO_4_ excretion. Hence, the present study on hypoparathyroid patients and normal population provides an opportunity to observe whether the phosphaturic effect of FGF-23 is independent of PTH or not.

In the present study, we detected high serum level of PO_4_ and FGF-23 in hypoparathyroid patients compared to the control group; however, we found no significant difference in FE PO_4_ or 1,25(OH)2 D level between the two groups. Also, we found a strong positive correlation between serum FGF-23 and FE PO_4_ in the control population, but this correlation was absent in hypoparathyroid patients. These findings could suggest that although the FGF-23 is one of the main regulator of urinary PO_4_ excretion, the existence of intact PTH is necessary for the full phosphaturic effect of FGF-23. However, further relevant human studies are warranted.

In the presence of normal parathyroid and kidney function inappropriate high serum FGF-23 could result in urinary PO_4_ loss and hypophosphatemia such as X-linked dominant hypophosphatemic rickets (XLH), [41–44], autosomal dominant hypophosphatemic rickets [45, 46], Autosomal recessive hypophosphatemic rickets (ARHR) [47, 48] or Fibrous dysplasia (FD)/McCune–Albright syndrome [49]. As well as some acquired disorders such as Tumor-induced osteomalacia (TIO) [50–52].

Previous studies showed that mean serum PO_4_ levels in hypoparathyroid patients remained above the normal range, even in the presence of high serum FGF-23 level [53].Yamashita et al. showed that in transient hypoparathyroidism high serum level of FGF23 and hyperphosphatemia will be normalized only after parathyroid recovery [54]. Animal studies also showed that PTH-null mice experienced high PO_4_ in spite of high circulating FGF-23, resembling participants with hypoparathyroidism in the present study [55]. In another study on hypoparathyroid patients, treatment with rhPTH could reduce serum PO_4_ level from the upper normal range to the normal values parallel with increased urinary PO_4_ excretion [56–58]. These findings also support our hypothesis about the importance of PTH in phosphaturic action of FGF-23. Another explanation by Gracia-Iguacel et al. was that PTH may have effect on phosphaturic function of FGF-23 through the serum calcium level [59]. Also, some studies showed that FGF23 have positive correlation with serum calcium [60, 61]. In our study, we found no correlation between calcium and FGF23 in low calcium and normal calcium level hypoparathyroid patients. However, the number of hypoparathyroid patients in low Ca group was just 7, which could affect the results. Another important issue in this regards was that normal renal function is necessary for this association. In some patients with chronic kidney disease, high serum phosphate was observed in spite of high serum PTH and FGF23 level [62]. Also, it was shown that in chronic kidney disease patients treated with hemodialysis, FGF23 could predict the progression of secondary hyperparathyroidism. Interestingly, in these patients total parathyroidectomy could decrease high serum FGF-23 level to normal values [63, 64].

In spite of many strengths of this study that evaluated FGF23 function in hypoparathyroid patients, we had some limitations. This study was a case-control cross sectional study, which could be better if we design an interventional clinical trial to evaluate the effect of PTH in hypoparathyroid patients in the future.

## Conclusion

The present study could suggest that although the FGF-23 is one of the main regulator of urinary PO_4_ excretion, the existence of sufficient parathyroid hormone is necessary for the full phosphaturic effect of FGF-23. We hypothesized that PTH might play a role in PO_4_ excretory signal pathway of FGF-23. However, further in vivo and in vitro studies are necessary to determine the mechanism of action of parathyroid hormone on PO_4_ excretory function of FGF-23.

## Data Availability

The datasets used and analyzed during the current study are available from the corresponding author on reasonable request.

## Abbreviations

1,25(OH)2 D: 1,25-dihydroxyvitamin D
25OHD: 25-hydroxy vitamin D
ALP: Alkaline phosphatase
Ca: Calcium
CKD: Chronic kidney diseases
DXA: Dual-energy X-ray absorptiometry
FE PO: Fractional excretion of phosphorous
FGF-23: Fibroblast growth factor 23
PTH: Parathyroid hormone
PO_4_: Phosphorus
SUMS: Shiraz University of Medical Sciences
NaPi II_a_ and II_c_: Sodium- phosphate cotransporters

## References

[1] Jüppner H (2011). Phosphate and FGF-23. Kidney Int.

[2] Miyamoto K-I, Haito-Sugino S, Kuwahara S, Ohi A, Nomura K, Ito M (2011). Sodium-dependent phosphate cotransporters: lessons from gene knockout and mutation studies. J Pharm Sci.

[3] Berndt T, Kumar R (2009). Novel mechanisms in the regulation of phosphorus homeostasis. Physiology..

[4] Kuro-o M (2008). Endocrine FGFs and Klothos: emerging concepts. Trends Endocrinol Metabolism.

[5] Berndt TJ, Schiavi S, Kumar R (2005). “Phosphatonins” and the regulation of phosphorus homeostasis. Am J Physiol-Renal Physiol.

[6] Murer H, Hernando N, Forster I (2000). Biber Jr. proximal tubular phosphate reabsorption: molecular mechanisms. Physiol Rev.

[7] Tenenhouse HS, Murer H (2003). Disorders of renal tubular phosphate transport. J Am Soc Nephrol.

[8] Fujii T, Shiozaki Y, Segawa H, Nishiguchi S, Hanazaki A, Noguchi M (2019). Analysis of opossum kidney NaPi-IIc sodium-dependent phosphate transporter to understand pi handling in human kidney. Clin Exp Nephrol.

[9] Farrow EG, White KE (2010). Recent advances in renal phosphate handling. Nat Rev Nephrol.

[10] Marks J, Debnam ES, Unwin RJ (2010). Phosphate homeostasis and the renal-gastrointestinal axis. Am J Physiol-Renal Physiol.

[11] Kaneko I, Tatsumi S, Segawa H (2017). Miyamoto K-i. control of phosphate balance by the kidney and intestine. Clin Exp Nephrol.

[12] Shimada T, Hasegawa H, Yamazaki Y, Muto T, Hino R, Takeuchi Y (2004). FGF-23 is a potent regulator of vitamin D metabolism and phosphate homeostasis. J Bone Miner Res.

[13] Shimada T, Kakitani M, Yamazaki Y, Hasegawa H, Takeuchi Y, Fujita T (2004). Targeted ablation of Fgf23 demonstrates an essential physiological role of FGF23 in phosphate and vitamin D metabolism. J Clin Invest.

[14] Fujii T, Segawa H, Hanazaki A, Nishiguchi S, Minoshima S, Ohi A et al. Role of the putative PKC phosphorylation sites of the type IIc sodium-dependent phosphate transporter in parathyroid hormone regulation. Clin Exp Nephrol. 2019;23:898–907. 10.1007/s10157-019-01725-6 30895530

[15] Weinman EJ, Biswas RS, Peng Q, Shen L, Turner CL, Xiaofei E (2007). Parathyroid hormone inhibits renal phosphate transport by phosphorylation of serine 77 of sodium-hydrogen exchanger regulatory factor–1. J Clin Invest.

[16] Shoback D (2008). Hypoparathyroidism. N Engl J Med.

[17] Bilezikian JP, Khan A, Potts JT, Brandi ML, Clarke BL, Shoback D (2011). Hypoparathyroidism in the adult: epidemiology, diagnosis, pathophysiology, target-organ involvement, treatment, and challenges for future research. J Bone Miner Res.

[18] Pajevic PD, Wein MN, Kronenberg HM. Parathyroid hormone actions on bone and kidney. Hypoparathyroidism. Milano: Springer; 2015. p. 99–109.

[19] Hadker N, Egan J, Sanders J, Lagast H, Clarke B (2014). Understanding the burden of illness associated with hypoparathyroidism reported among patients in the paradox study. Endocr Pract.

[20] Marx SJ (2000). Hyperparathyroid and hypoparathyroid disorders. N Engl J Med.

[21] Underbjerg L, Sikjaer T, Mosekilde L, Rejnmark L (2013). Cardiovascular and renal complications to postsurgical hypoparathyroidism: a Danish nationwide controlled historic follow-up study. J Bone Miner Res.

[22] Powers J, Joy K, Ruscio A, Lagast H (2013). Prevalence and incidence of hypoparathyroidism in the United States using a large claims database. J Bone Miner Res.

[23] Abate EG, Clarke BL (2017). Review of hypoparathyroidism. Front Endocrinol.

[24] Berndt T, Kumar R (2007). Phosphatonins and the regulation of phosphate homeostasis. Annu Rev Physiol.

[25] Imel EA, Econs MJ (2005). Fibroblast growth factor 23: roles in health and disease. J Am Soc Nephrol.

[26] Quarles LD (2008). Endocrine functions of bone in mineral metabolism regulation. J Clin Invest.

[27] Tan SJ, Smith ER, Hewitson TD, Holt SG, Toussaint ND (2014). The importance of klotho in phosphate metabolism and kidney disease. Nephrology..

[28] Strom TM, Jüppner H (2008). Phex, FGF23, DMP1 and beyond. Curr Opin Nephrol Hypertens.

[29] Andrukhova O, Zeitz U, Goetz R, Mohammadi M, Lanske B, Erben RG (2012). FGF23 acts directly on renal proximal tubules to induce phosphaturia through activation of the ERK1/2–SGK1 signaling pathway. Bone..

[30] Farrow EG, Davis SI, Summers LJ, White KE (2009). Initial FGF23-mediated signaling occurs in the distal convoluted tubule. J Am Soc Nephrol.

[31] Kuro-o M (2017). The FGF23 and Klotho system beyond mineral metabolism. Clin Exp Nephrol.

[32] Chang Q, Hoefs S, Van Der Kemp A, Topala C, Bindels R, Hoenderop J (2005). The ß-glucuronidase klotho hydrolyzes and activates the TRPV5 channel. Science..

[33] Cole JA (1999). Parathyroid hormone activates mitogen-activated protein kinase in opossum kidney cells. Endocrinology..

[34] de Groot T, Lee K, Langeslag M, Xi Q, Jalink K, Bindels RJ (2009). Parathyroid hormone activates TRPV5 via PKA-dependent phosphorylation. J Am Soc Nephrol.

[35] Lavi-Moshayoff V, Wasserman G, Meir T, Silver J, Naveh-Many T (2010). PTH increases FGF23 gene expression and mediates the high-FGF23 levels of experimental kidney failure: a bone parathyroid feedback loop. Am J Physiol-Renal Physiol.

[36] Imura A, Tsuji Y, Murata M, Maeda R, Kubota K, Iwano A (2007). α-Klotho as a regulator of calcium homeostasis. Science..

[37] Ben-Dov IZ, Galitzer H, Lavi-Moshayoff V, Goetz R, Kuro-o M, Mohammadi M (2007). The parathyroid is a target organ for FGF23 in rats. J Clin Invest.

[38] Krajisnik T, Björklund P, Marsell R, Ljunggren O, Akerström G, Jonsson KB (2007). Fibroblast growth factor-23 regulates parathyroid hormone and 1-hydroxylase expression in cultured bovine parathyroid cells. J Endocrinol.

[39] Canalejo R, Canalejo A, Martinez-Moreno JM, Rodriguez-Ortiz ME, Estepa JC, Mendoza FJ (2010). FGF23 fails to inhibit uremic parathyroid glands. J Am Soc Nephrol.

[40] Andrukhova O, Streicher C, Zeitz U, Erben RG (2016). Fgf23 and parathyroid hormone signaling interact in kidney and bone. Mol Cell Endocrinol.

[41] Jonsson KB, Zahradnik R, Larsson T, White KE, Sugimoto T, Imanishi Y (2003). Fibroblast growth factor 23 in oncogenic osteomalacia and X-linked hypophosphatemia. N Engl J Med.

[42] Yamazaki Y, Okazaki R, Shibata M, Hasegawa Y, Satoh K, Tajima T (2002). Increased circulatory level of biologically active full-length FGF-23 in patients with hypophosphatemic rickets/osteomalacia. J Clin Endocrinol Metabolism.

[43] Miyamoto K-I, Taketani Y, Morita K, Segawa H, Nii T, Fujioka A (1998). Molecular and cellular regulation of renal phosphate transporters in X-linked hypophosphatemia. Clin Exp Nephrol.

[44] Nii T, Taketani Y, Tani Y, Ohkido I, Segawa H, Yamamoto H (2001). Direct demonstration of humorally mediated inhibition of the transcription of phosphate transporter in XLH patients. Clin Exp Nephrol.

[45] Shimada T, Muto T, Urakawa I, Yoneya T, Yamazaki Y, Okawa K (2002). Mutant FGF-23 responsible for autosomal dominant hypophosphatemic rickets is resistant to proteolytic cleavage and causes hypophosphatemia in vivo. Endocrinology..

[46] Imel EA, Hui SL, Ecibs MJ (2007). FGF23 concentrations vary with disease status in autosomal dominant hypophosphatemic rickets. J Bone Miner Res.

[47] Levy-Litan V, Hershkovitz E, Avizov L, Leventhal N, Bercovich D, Chalifa-Caspi V (2010). Autosomal-recessive hypophosphatemic rickets is associated with an inactivation mutation in the ENPP1 gene. Am J Hum Genet.

[48] Lorenz-Depiereux B, Schnabel D, Tiosano D, Häusler G, Strom TM (2010). Loss-of-function ENPP1 mutations cause both generalized arterial calcification of infancy and autosomal-recessive hypophosphatemic rickets. Am J Hum Genet.

[49] Riminucci M, Collins MT, Fedarko NS, Cherman N, Corsi A, White KE (2003). FGF-23 in fibrous dysplasia of bone and its relationship to renal phosphate wasting. J Clin Invest.

[50] Shimada T, Mizutani S, Muto T, Yoneya T, Hino R, Takeda S (2001). Cloning and characterization of FGF23 as a causative factor of tumor-induced osteomalacia. Proc Natl Acad Sci.

[51] Folpe AL, Fanburg-Smith JC, Billings SD, Bisceglia M, Bertoni F, Cho JY (2004). Most osteomalacia-associated mesenchymal tumors are a single histopathologic entity: an analysis of 32 cases and a comprehensive review of the literature. Am J Surg Pathol.

[52] Rowe PS (1998). X-linked rickets and tumor-acquired osteomalacia: PHEX and the missing link. Clin Exp Nephrol.

[53] Gupta A, Winer K, Econs M, Marx S, Collins M (2004). FGF-23 is elevated by chronic hyperphosphatemia. J Clin Endocrinol Metabolism..

[54] Yamashita H, Yamazaki Y, Hasegawa H, Yamashita T, Fukumoto S, Shigematsu T, Kazama JJ, Fukagawa M, Noguchi S. Fibroblast growth factor-23 (FGF23) in patients with transient hypoparathyroidism: its important role in serum phosphate regulation. Endocr J. 2007;54(3):465-70. 10.1507/endocrj.k06-156 17464094

[55] Bai X, Miao D, Goltzman D, Karaplis AC (2007). Early lethality in Hyp mice with targeted deletion of Pth gene. Endocrinology..

[56] Clarke BL, Vokes TJ, Bilezikian JP, Shoback DM, Lagast H, Mannstadt M (2017). Effects of parathyroid hormone rhPTH (1–84) on phosphate homeostasis and vitamin D metabolism in hypoparathyroidism: REPLACE phase 3 study. Endocrine..

[57] Clarke BL, Berg JK, Fox J, Cyran JA, Lagast H (2014). Pharmacokinetics and pharmacodynamics of subcutaneous recombinant parathyroid hormone (1–84) in patients with hypoparathyroidism: an open-label, single-dose, phase I study. Clin Ther.

[58] Sikjaer T, Rejnmark L, Rolighed L, Heickendorff L, Mosekilde L, Group HS (2011). The effect of adding PTH (1–84) to conventional treatment of hypoparathyroidism: a randomized, placebo-controlled study. J Bone Miner Res.

[59] Gracia-Iguacel C, Gonzalez-Parra E, Rodriguez-Osorio L, Sanz AB, Almaden Y, de la Piedra C, Egido J, Rodriguez M, Ortiz A. Correction of hypocalcemia allows optimal recruitment of FGF-23-dependent phosphaturic mechanisms in acute hyperphosphatemia post-phosphate enema. J Bone Miner Metab. 2013;31(6):703-7. 10.1007/s00774-013-0435-z 23677707

[60] Rodriguez-Ortiz ME, Lopez I, Muñoz-Castañeda JR, Martinez-Moreno JM, Ramírez AP, Pineda C, Canalejo A, Jaeger P, Aguilera-Tejero E, Rodriguez M, Felsenfeld A. Calcium deficiency reduces circulating levels of FGF23. J Am Soc Nephrol. 2012;23(7):1190-7. 10.1681/ASN.2011101006 PMC3380648 22581996

[61] Yamashita H, Yamashita T, Miyamoto M, Shigematsu T, Kazama JJ, Shimada T, Yamazaki Y, Fukumoto S, Fukagaw M, Noguchi S. Fibroblast growth factor (FGF)-23 in patients with primary hyperparathyroidism. Eur J Endocrinol. 2004;151(1):55-60. 10.1530/eje.0.1510055 15248822

[62] Ramon I, Kleynen P, Body JJ, Karmali R. Fibroblast growth factor 23 and its role in phosphate homeostasis. Eur J Endocrinol. 2010;162(1):1. 10.1530/EJE-09-0597 19776202

[63] Nakanishi S, Kazama JJ, Nii-Kono T, Omori K, Yamashita T, Fukumoto S, Gejyo F, Shigematsu T, Fukagawa M. Serum fibroblast growth factor-23 levels predict the future refractory hyperparathyroidism in dialysis patients. Kidney Int. 2005;67(3):1171-8. 10.1111/j.1523-1755.2005.00184.x 15698459

[64] Sato T, Tominaga Y, Ueki T, Goto N, Matsuoka S, Katayama A, Haba T, Uchida K, Nakanishi S, Kazama JJ, Gejyo F. Total parathyroidectomy reduces elevated circulating fibroblast growth factor 23 in advanced secondary hyperparathyroidism. Am J Kidney Dis. 2004;44(3):481-7. 15332221

